# Utilization and Dose Optimization of Angiotensin‐Converting Enzyme Inhibitors in Heart Failure Patients With Reduced Ejection Fraction: A Cross Sectional Study on Implications for Guideline‐Targeted Therapy

**DOI:** 10.1002/hsr2.71451

**Published:** 2025-11-06

**Authors:** Martin Kampamba, Ruth Mbanvu, Jimmy Hangoma, Gunet Mwalungali, Mukumbi Mutenda, Audrey Hamachila

**Affiliations:** ^1^ Department of Pharmacy, School of Health Sciences University of Zambia Lusaka Zambia; ^2^ Department of Pharmacy, School of Health Sciences Levy Mwanawasa Medical University Lusaka Zambia; ^3^ School of Medicine Cavendish University Lusaka Zambia; ^4^ Department of Pharmacy Children′s Hospital, University Teaching Hospitals Lusaka Zambia

**Keywords:** dose optimization, heart failure, reduced ejection fraction, utilization

## Abstract

**Background and Aims:**

Angiotensin‐converting enzyme inhibitors (ACEIs) are well established in reducing morbidity and mortality among patients with chronic heart failure (CHF). Evidence has consistently supported the use of high‐dose ACEI therapy, which is recommended to optimize clinical outcomes and enhance patients' quality of life. Suboptimal utilization and under‐dosing of these agents are associated with increased rates of hospitalization, worsening symptoms of heart failure, and overall, poor prognoses. We assessed the utilization and dose optimization of ACEIs in heart failure patients at the Adult University Teaching Hospital (AUTH).

**Methods:**

This was a cross‐sectional study conducted among adult patients with with a confirmed diagnosis of CHF and an ejection fraction of < 40%. A checklist was used to collect data, which was subsequently analysed using STATA version 15.1. Multivariable logistic regression was used to determine associated factors.

**Results:**

Of the 292 study participants, 209 (71.6%) were on ACEIs, with Enalapril being the only drug in use. The study found that only 42 (14.6%) of the patients were taking the optimum doses. The adjusted logistic regression model revealed that New York Heart Association (NYHA) class II (AOR: 3.1, CI: 1.20–8.01, *p* = 0.018), NYHA class III (AOR: 1.6, CI: 0.72–13.7, *p* = 0.004), and those taking at least 5 medications (AOR: 1.9, CI: 1.17–3.04, *p* = 0.010) were more likely to utilize ACEIs. Diabetes mellitus (COR: 4.3, CI: 1.38–13.3, *p* = 0.012) and having hypertension (COR: 6.2, CI: 2.15–17.9 *p* = 0.001) were significantly associated with higher likelihood of dose optimization of ACEIs. Hypertension was the most common comorbidity, affecting 183 (62.5%) participants while the majority, 176 (60.4%) of the individuals had less than two comorbidities.

**Conclusion:**

Although the majority of patients were receiving ACEIs, only a small proportion of these were using optimal dosages. Therefore, a multidisciplinary team approach that includes clinical pharmacists in the medication review and patient monitoring processes would achieve definite outcomes in patients with HF.

## 1 Background

Heart failure (HF) is a significant global health issue, affecting approximately 26 million people worldwide, with a higher prevalence among the elderly population [1, 2]. HF is becoming an increasingly important public health concern in developing regions, including sub‐Saharan Africa [3]. It is a debilitating condition associated with high rates of morbidity and mortality, reduced quality of life, and increased healthcare costs [4, 5]. Despite substantial therapeutic advancements in the management of HF over recent decades, it remains a leading cause of morbidity, mortality, and economic strain on healthcare systems [6].

It is well known that renin‐angiotensin‐aldosterone system (RAAS) is a crucial therapeutic target in managing HF patients [7, 8]. Recent clinical trials have demonstrated that the use of angiotensin‐converting enzyme inhibitors (ACEIs) and angiotensin II receptor blockers (ARBs) significantly reduces morbidity and mortality in cardiovascular events among HF patients [9, 10, 11]. Studies have also consistently shown that ACEI therapy alleviates symptoms, decreases hospitalizations, and improves survival rates in HF patients presenting with reduced ejection fraction [8, 12].

Clinical evidence suggests that HF patients benefit from ACEIs when administered in a dose‐dependent manner, with higher target doses yielding more substantial outcomes [13, 14]. Upward titration of ACEIs to the maximum tolerable dose is essential in reducing hospital admissions, lower mortality rates, and enhance their quality of life [15, 16, 17]. Several studies have confirmed that the majority of patients with chronic HF can achieve optimal doses of ACEIs to experience better clinical outcomes [12, 18]. Therefore, achieving the target dose is critical for maximizing clinical benefits [19].

According to evidence‐based guidelines [19, 20], the recommended daily target doses of ACEIs for HF management include 20–40 mg enalapril, 10 mg ramipril, 150 mg captopril, 20–40 mg lisinopril, 40 mg fosinopril, 4 mg trandolapril, 40 mg quinapril, or 8–16 mg perindopril [17, 21, 22]. Studies suggest that these target doses are tolerable for the majority of HF patients when titrated appropriately [23]. However, some research found that many patients receive lower doses than recommended. For instance, a study conducted in Ethiopia revealed that while most patients were prescribed ACEIs, only about one‐third received optimal dosages [6]. Similar findings have been reported in other studies [14, 24, 25]. Despite the wealth of research on ACEI optimization in various countries, no studies have yet explored this in Zambia. Therefore, this study aimed to investigate the utilization and optimization of ACEI therapy and associated factors in managing ambulatory HF patients at the Adult University Teaching Hospital (AUTH).

## 2 Methods and Materials

### 2.1 Study Design, Setting and Population

This was a cross‐sectional study conducted among heart failure patients aged 18 years and older at the Adult University Teaching Hospitals in the Department of Internal Medicine in Lusaka.

### 2.2 Inclusion Criteria

The study included patients aged at least 18 years old diagnosed with heart failure with an ejection fraction of less than 40% (confirmed by echocardiography), on treatment with at least one ACEI, with or without additional medications for a minimum follow‐up period of 6 months.

### 2.3 Exclusion Criteria

Patients were excluded if they had conditions where the use of ACEIs is contraindicated or requires precautions. These included a history of angioedema, pregnancy, an allergic reaction to ACEIs, persistent dry cough, hyperkalemia (> 5.5 meq/ml), hypotension (< 90/60 mmHg), renal insufficiency (creatinine clearance < 30 mL/min), aortic or mitral stenosis, bilateral renal artery stenosis, and incomplete medical records. Additionally, patients with missing key predictor variables were excluded.

### 2.4 Data Collection Technique

#### 2.4.1 Review of Patient′S Drug Chart and Files

The data collection process involved a review of patients' drug charts and medical files. Secondary data was used, and a data extraction checklist was designed to facilitate the collection of relevant information. The drug charts and files of both inpatients and outpatients were screened for ACEIs use and dose optimization. This screening included verifying that prescriptions on the medication charts were consistent with those in the medical records and ensuring the clarity of the prescription charts.

Sociodemographic, clinical, and treatment‐related characteristics were extracted from patients' medical records using a data abstraction checklist. The study assessed the utilization and dosing of ACEIs. ACEI use was considered suboptimal if the patient was not prescribed ACEIs despite the absence of contraindications. Optimal dosing of ACEIs was determined based on guideline‐recommended target doses (Yancy, 2013). A dose was considered optimal if the patient received the target dose or the maximum tolerable dose. For enalapril, an optimal dose was defined as the maximum tolerable dose of ≥ 20 mg daily up to 40 mg. For captopril, the optimal dose was defined as ≥ 150 mg daily or the maximum tolerable dose. Similarly, for lisinopril, the target was 20–40 mg daily; 40 mg daily for fosinopril, 4 mg daily for trandolapril, 40 mg daily for quinapril and for perindopril, 8–16 mg daily. Doses were categorized as suboptimal if they were below the target dose without contraindications for up‐titration [23]. The study utilized simple random sampling to select patient files for review.

#### 2.4.2 Sample Size Determination

The single population proportion formula (*n* = *z*²*p* (1 − *p*)/*d*²) was used to estimate the sample size as reported from a hospital based cross‐sectional study which reported 74.7% utilization of ACE‐Is among heart failure patients [6]. Where *n* = sample size, *z* = 95% confidence level at 1.96, *d* = margin of error (*n* = 1.96² × 0.747 (1 − 0.747)/0.05², *n* = 292 patients. 292 participants were enrolled for the study.

### 2.5 Data Analysis

The data were checked and cleaned on a daily basis during collection and before analysis. STATA version 15.1 (Stata Corp, USA) was used to analyse the collected data. Descriptive statistics such as means, frequencies and percentages were used to examine utilization and dose optimization of ACEIs. The Shapiro‐Wilk test was used to test for normality, and continuous variables with a *p* > 0.05 were considered to be normally distributed. For parametric data, the mean and standard deviation (SD) were computed, while median and interquartile range (IQR) were computed for non‐parametric data. We further performed a bivariate logistic regression analysis to determine the association of each independent variable with utilization and optimization of ACE‐Is. All independent variables with a *p* < 0.2 in bivariate analysis were incorporated in the multivariable logistic regression model to identify factors independently associated with utilization and dose optimization of ACE‐Is. A *p* value (two‐tailed) of less than 0.05 was considered to indicate statistical significance in all analyses. Findings from data analysis were described in text and presented in tables and figures.

### 2.6 Ethical Approval

Permission to conduct this study was sought from the University of Zambia, School of Health Sciences Research Ethics Committee (UNZA‐SHSREC) which was granted with protocol ID number 202112030063. Confidentiality about the information obtained during the study was assured and maintained. Names were not disclosed but codes were used to enter the data.

## 3 Results

### 3.1 Sociodemographic Characteristics of Participants

The sociodemographic results (Table 1) indicated that the majority of the 292 participants were female, 166 (57%). Most 229 (78.4%) participants were under the age of 65 and a significant majority, 254 (87%), resided in urban areas. In terms of education, the largest group, 158 (53.9%), had attained secondary education. Regarding marital status, most 192 (65.6%) participants, were married.

**Table 1:** Sociodemographic characteristics of the participants (*n* = 292).

Variable	Category	Frequency (%)
Sex	Male	126 (43)
	Female	166 (57)
Age	< 65	229 (78.4)
	≥ 65	63 (21.6)
Residence	Rural	38 (13)
	Urban	254 (87)
Educational level	Primary	65 (22.1)
	Secondary	158 (53.9)
	Tertiary	69 (24)
Marital Status	Single	37 (12.5)
	Married	192 (65.6)
	Divorced	4 (1.3)
	Widowed	59 (20.6)

### 3.2 Clinical and Medication Related Characteristics

The clinical and medication‐related characteristics of the participants revealed that a majority, 211 (72.4%), had previous hospitalizations. Most participants, 159 (73.3%), had a duration of hospitalization of more than 2 weeks. Regarding the NYHA classification, the majority, 214 (73.4%), were classified as Class III. The median ejection fraction (EF) was 30% (IQR: 25, 37). The mean systolic and diastolic blood pressure was 125 mmHg (SD: 19.6) and 80 mmHg (SD: 14) respectively, while the median heart rate was 91 bpm (IQR: 81, 100). The most frequently identified comorbidities included 183 (62.5%) hypertension and 163 (55.7%) dilated cardiomyopathy. A majority of participants, 176 (60.4%), had fewer than two comorbidities. Additionally, most participants, 181 (62%), were on five or more prescription medications (Table 2).

**Table 2:** Clinical and medication related characteristics of the participants.

Characteristics		Frequency (%)
Previous hospitalization	No	81 (27.6)
	Yes	211 (72.4)
Duration of hospitalization (*n* = 292)	< 2 weeks	159 (75.3)
	≥ 2 weeks	52 (17.7)
NYHA class	I	25 (8.6)
	II	53 (18)
	III	214 (73.4)
Ejection fraction	EF median (IQR)	30 (25, 37)
Blood pressure	Systolic BP mean (SD)	125 (19.6)
	Diastolic BP mean (SD)	8 (14)
	Heart rate median (IQR)	91 (81, 100)
FrequentlyIdentified comorbidities	Hypertension	183 (62.5)
	Dilated cardiomyopathy	163 (55.7)
	Diabetes mellitus	27 (9.1)
	Chronic kidney disease	18 (6)
Number of comorbidities	< 2	176 (60.4)
	≥ 2	116 (39.6)
Number of medications	< 5	111 (38)
	≥ 5	181 (62)

### 3.3 Utilization and Dose Optimization of ACEIs in HF Patients

Out of a total of 292 patients, 209 (71.6%) were receiving ACEIs. Enalapril was the only prescribed ACEI and only 43 (14.6%) of the patients were taking optimal doses. A total of 57 (19.6%) patients were receiving doses between 50% and less than 100% of the target dose (20 mg to under 40 mg), while 181 (65.8%) patients were on doses below 50% of the target dose (< 20 mg). The minimum dose used was 2.5 mg while the maximum was 40 mg. Furthermore, the median dose used was 5 mg, with an interquartile range between 2.5 mg and 10 mg (Table 3).

**Table 3:** Summary of the type and dose of ACEIs used in CHF patients (*n* = 292).

Variables	Enalapril medication; Frequency (%)
Number of patients on the medication	209 (71.6)
Number of patients on the optimal dose	43 (14.6)
Number of patients on 50 to < 100% of the target dose	57 (19.6)
Number of patients on < 50% of the target dose	192 (65.8)
Median (IQR) dose received (mg)	5 (2.5, 10)
Minimum dose used (mg/d)	2.5
Maximum dose used (mg/d)	40

### 3.4 Commonly Used Medications in Congestive Heart Failure Patients

**Figure 1:**
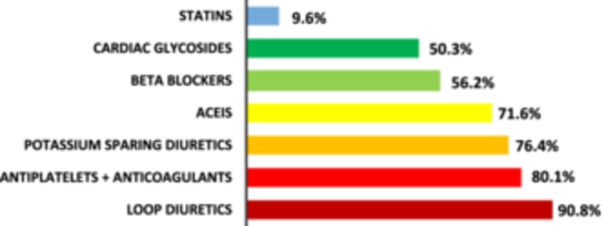
Commonly used medications (*n* = 292).

As shown in Figure 1, the most commonly utilized medications were loop diuretics, 265 (90.8%); antiplatelets and anticoagulants, 234 (80.1%); potassium‐sparing diuretics, 223 (76.4%); and ACE inhibitors, 209 (71.6%).

### 3.5 Factors Associated With Utilization of ACEIs in CHF

In the unadjusted logistic regression model, NYHA Class II (COR: 3.7, 95% CI: 1.50–9.10, *p* = 0.004), Class III (COR: 2.1, 95% CI: 1.04–4.44, *p* = 0.04), and the use of five or more medications (COR: 2.0, 95% CI: 1.31–3.19, *p* = 0.002) were significantly associated with a higher likelihood of ACEIs dose utilization. In the adjusted logistic regression model, previous hospitalization within the year (AOR: 2.0, CI: 1.13–3.42, *p* = 0.017) was significantly associated with higher likelihood of ACEIs utilization, while NYHA Class II (AOR: 3.1, 95% CI: 1.20–8.01, *p* = 0.02), Class III (AOR: 1.6, 95% CI: 0.72–13.7, *p* = 0.004), and the use of five or more medications (AOR: 1.9, 95% CI: 1.17–3.04, *p* = 0.01) were significantly associated with a higher likelihood of ACEIs utilization (Table 4).

**Table 4:** Unadjusted and adjusted binary logistic regression models examining the factors associated with utilization of ACE‐Inhibitors.

Variable	Category	Unadjusted			Adjusted		
		COR	95% CI	*p* value	AOR	95% CI	*p* value
Gender	Male	1					
	Female	0.7	0.48, 1.15	0.19*	0.7	0.43, 1.18	0.20
Age	< 65	1					
	≥ 65	1.9	1.06, 3.41	0.03*	1.7	0.92, 3.15	0.09
Previous hospitalization within the year	No	1					
	Yes	1.5	0.94, 2.44	0.08*	2.0	1.13, 3.42	0.017**
NYHA classification	Class I	1					
	Class II	3.7	1.50, 9.10	0.004	3.1	1.20, 8.01	0.02**
	Class III	2.1	1.04, 4.44	0.04*	1.6	0.72, 13.7	0.004**
Number of comorbidity	< 2	1					
	≥ 2	0.8	0.50, 1.20	0.25	0.9	0.49, 3.38	0.26
Number of medication	< 5	1					
	≥ 5	2.0	1.31, 3.19	0.002*	1.9	1.17, 3.04	0.01**
Daily dose of furosemide	< 40 mg	1					
	≥ 40 mg	0.8	0.48, 1.25	0.30	—	—	—
Presence of diabetes mellitus	No	1					
	Yes	1.8	0.77, 2.76	0.17	1.2	0.52, 2.56	0.72

Abbreviations: 1, reference category; AOR, adjusted odds ratio; CI, confidence interval; OR, crude odds ratio.

* *p* < 0.05 in adjusted model

** *p* < 0.2 in unadjusted model.

### 3.6 Factors Associated With Dose Optimization of ACEIs in CHF Patients

In the unadjusted logistic regression model, being female (COR: 0.3, 95% CI: 0.16–0.67, *p* = 0.0001) was significantly associated with a lower likelihood of dose optimization of ACEIs. In contrast, having two or more comorbidities (COR: 2.0, 95% CI: 1.04–3.89, *p* = 0.038), diabetes mellitus (COR: 2.9, 95% CI: 1.23–7.00, *p* = 0.015), and hypertension (COR: 4.7, 95% CI: 1.81–12.4, *p* = 0.001) were significantly associated with a higher likelihood of ACEI dose optimization. In other words, heart failure patients with diabetes were more likely to have ACEIs optimized than nondiabetic patients, and those with hypertension were more likely to have ACEIs optimized than non‐hypertensive patients.

In the adjusted logistic regression model, being female (AOR: 0.2, 95% CI: 0.09–0.40, *p* = 0.0001) remained significantly associated with a lower likelihood of ACEI dose optimization, implying that female patients with heart failure were less likely to achieve ACEI dose optimization compared to their male counterparts. Additionally, diabetes mellitus (AOR: 4.3, 95% CI: 1.38–13.3, *p* = 0.012) and hypertension (AOR: 6.2, 95% CI: 2.15–17.9, *p* = 0.001) were independently associated with a higher likelihood of ACEI dose optimization. This implies that the presence of diabetes and/or hypertension in heart failure patients increases the odds of ACEI dose optimization by approximately 4.3 and 6.2 times, respectively (Table 5).

**Table 5:** Unadjusted and adjusted binary logistic regression models examining the factors.

Associated with dose optimization of ACEIs variable	Category	Unadjusted			Adjusted		
		COR	95% CI	*p* value	AOR	95% CI	*p* value
Gender	Male	1					
	Female	0.3	0.16, 0.67	0.001**	0.2	0.09, 0.40	0.0001*
Age	< 65	1					
	≥ 65	1.4	0.68, 3.00	0.34	—	—	—
Previous hospitalization within the year	No	1					
	Yes	0.7	0.33, 1.35	0.27	—	—	—
NYHA classification	Class I	1					
	Class II	1.4	0.65, 3.07	0.37	—	—	—
	Class III	1.7	0.74, 4.44	0.41	—	—	—
Number of comorbidity	< 2	1					
	≥ 2	2.0	1.04, 3.89	0.038**	0.8	0.27, 1.74	0.53
Number of medication	< 5	1					
	≥ 5	0.6	0.33, 1.25	0.19**	0.6	0.28, 1.20	0.14
Daily Dose of Furosemide	< 40 mg	1					
	≥ 40 mg	1.1	0.56, 2.30	0.72	—	—	—
Presence of diabetes mellitus	No	1					
	Yes	2.9	1.23, 7.00	0.015**	4.3	1.38, 13.3	0.01*
Presence of hypertension	No	1					
	Yes	4.7	1.81,12.4	0.001**	6.2	2.15,17.9	0.001*

Abbreviations: 1, reference category; AOR, adjusted odds ratio; CI, confidence interval; OR, crude odds ratio.

* *p* < 0.05 in adjusted model

** *p* < 0.2 in unadjusted model.

## 4 Discussion

Chronic heart failure (CHF) is among the leading causes of mortality worldwide, placing a significant economic burden on healthcare systems [26]. Therefore, optimizing CHF therapy is crucial [27]. Over the past 20 years, numerous trials involving patients with CHF have concluded that the use of ACEIs confers a 16% to 20% reduction in mortality [28]. In this study, the utilization and optimization of ACEIs and their associated factors were evaluated.

A total of 292 patients' files were reviewed, revealing that 71.6% of patients had utilized ACEIs, with Enalapril being the only ACEI in use at the time of the study. This finding is consistent with studies from Ethiopia, Palestine, India and German, where utlisation rates were 74.7%, 70.1%, 76.7%, and 76% respectively [6, 29, 30, 31]. According to guideline‐targeted therapy, all patients with systolic heart failure with reduced ejection fraction (HFrEF) should be prescribed ACEIs unless contraindicated [21, 32, 33]. However, several studies, including our study, have reported underutilization of ACEIs despite the absence of contraindications [30, 34, 35]. Addressing this underutilization is critical for improving patient outcomes and aligning practice with established guidelines, especially, given that clinical trial evidence has shown that ACEIs reduce both morbidity and mortality in patients with HFrEF [36, 37]. In the current study, while NYHA Class II and Class III were significantly associated with a higher likelihood of ACEIs utilization. This is in contrast with findings from previous studies, where NYHA Class III was not associated with ACEI utilization [6, 21]. Differences in patient preferences, or the biases and prescribing habits of healthcare providers, could influence ACEI utilization. Additionally, the study found that patients taking five or more medications were more likely to utilize ACEIs. This may reflect the presence of additional comorbidities such as hypertension and diabetes mellitus, which often warrant ACEI use as part of a comprehensive treatment plan [38, 39]. Despite an extensive literature review, no previous studies were identified with similar findings.

Studies indicate that using the maximum tolerable doses of ACEIs significantly reduces both morbidity and mortality [40, 41]. It is against such findings that evidence‐based guidelines recommend titrating ACEIs to the target dose, unless tolerability issues arise [21, 32, 33]. In the current study, only 14.6% of patients on ACEIs were receiving optimal doses, indicating that ACEIs were under‐dosed in the majority of heart failure patients. This finding aligns with reports from Tigray, Ethiopia (27.8%), Bahir Dar, Ethiopia (30.6%), and several other studies [6, 14, 34]. Conversely, higher rates of optimal dosing have been reported in Germany (62%) and Palestine (51%) [30, 31]. Despite these variations, all patients with HFrEF should be prescribed ACEIs unless contraindicated, underscoring the importance of adhering to guideline‐recommended treatment strategies.

In the present study, being female was significantly associated with a lower likelihood of dose optimization of ACEIs. This finding contrasts with a study conducted in Palestine, where sociodemographic characteristics such as gender were not associated with dose optimization [30]. The discrepancy may be attributed to differences in healthcare systems, cultural attitudes towards gender, or variations in the clinical management of heart failure between the two regions. Additionally, differences in sample size, study design, and statistical methods could also contribute to the divergent findings. In our study, age was not associated with ACEI dose optimization. However, two separate studies conducted in Ethiopia found that patients aged ≥ 65 were more likely to receive suboptimal doses [6, 42]. This contrast may be due to variations in population demographics, with differing baseline characteristics and comorbidities influencing prescribing practices in older adults. Furthermore, regional differences in healthcare infrastructure, access to medical resources, and clinician experience may also impact how age‐related factors are considered in dose optimization.

In the present study, having diabetes mellitus was significantly associated with a higher likelihood of ACEI dose optimization. This finding contrasts with reports from other studies, where diabetes mellitus was not associated with dose optimization [6, 30]. Several factors may contribute to this discrepancy. Firstly, differences in study populations could play a significant role. Our study may have included a higher proportion of patients with diabetes who underwent more frequent clinical follow‐ups or closer monitoring due to their diabetic condition. Enhanced monitoring may lead to more aggressive management and optimization of ACEI doses. Secondly, healthcare providers in our setting might be more attuned to the benefits of optimizing ACEI therapy in diabetic patients, recognizing the dual benefit of managing both heart failure and diabetic complications effectively.

Additionally, the current study revealed that having hypertension was significantly associated with ACEI dose optimization. This finding is supported by a study conducted in Palestine [30]. In both studies, hypertension was among the most frequently identified comorbidities. Furthermore, clinical guidelines often emphasize the use of ACEIs in the management of both hypertension and HFrEF [43]. These dual indications may prompt clinicians to be more vigilant in ensuring that patients with hypertension receive optimal doses to achieve therapeutic goals for both conditions. Optimizing ACEI therapy can lead to better blood pressure control and improved heart failure outcomes [44], providing a strong rationale for clinicians to prioritize dose optimization.

### 4.1 Limitations

This was an institutional based cross‐sectional study at a tertiary hospital which is a government institution. Therefore, differences in clinical practices, patient demographics, and healthcare personnel and infrastructure could limit the applicability of the results to broader contexts. Additionally, the retrospective nature of the study may introduce bias, as it relies on the accuracy and completeness of existing medical records. Information may have been incorrectly recorded, affecting the study′s findings. However, missing records were excluded from the study. It is possible that the percentage of patients using optimal doses was underestimated. For instance, some patients may have previously attempted higher doses but had to discontinue due to intolerance. This retrospective analysis may not fully capture those dose adjustments and the reasons behind them, potentially leading to an underestimation of the actual efforts made to optimize doses. This study excluded patients with renal insufficiency (creatinine clearance < 30 mL/min), which may limit the generalizability of the findings to patients with advanced chronic kidney disease or those receiving dialysis. Given the increasing interest in the use of RAAS inhibitors in high‐risk populations, future studies should explore ACEI optimization in patients with impaired renal function to inform clinical decision‐making.

## 5 Conclusion

Although majority of patients were receiving ACEIs, enalapril was the only ACEI in use and only about 14.6% of these were using optimal dosage. Diabetes mellitus and NYHA III were positively associated with the use of ACEIs whereas NYHA I was negatively associated. Furthermore, the presence of hypertension and dilated cardiomyopathy were significantly associated with suboptimal dosing of enalapril. Therefore, implementation of multidisciplinary team approach in the medication review and patient monitoring process for the optimization of ACEIs and achieving definite outcomes in patients with HF would be of great benefit. Furthermore, efforts need to be made to minimize potentially modifiable risk factors of suboptimal use of ACEIs in HF patients.

## Author Contributions

Martin Kampamba, Ruth Mbanvu, and Audrey Hamachila contributed to designing, supervising, and coordinating the project. Ruth Mbanvu and Martin Kampamba contributed to material preparation and data collection. Martin Kampamba analysed the data. Martin Kampamba, Jimmy Hangoma and Gunet Mwalungali contributed to the interpretation of the analyses of data. Mukumbi Mutenda and Martin Kampamba supervised the project and were responsible for quality control. The first draft of the manuscript was written by Martin Kampamba, Ruth Mbanvu and Audrey Hamachila. All authors commented on the previous versions of the manuscript and approved the final manuscript.

## Ethical Statement

The protocol for this study was approved by the University of Zambia School of Health Sciences Research Ethics Committee (Study protocol ID: 202112030063). We obtained informed, written consent from all participants before the study. The participants' confidentiality and anonymity were strictly maintained.

## Conflicts of Interest

The authors declare no conflicts of interest.

## Transparency Statement

The lead author Martin Kampamba affirms that this manuscript is an honest, accurate, and transparent account of the study being reported; that no important aspects of the study have been omitted; and that any discrepancies from the study as planned (and, if relevant, registered) have been explained.

## Data Availability

Upon reasonable request, the corresponding author, M.K., would provide the data that support the study′s conclusions.
